# Integrative gene expression analysis and animal model reveal immune‐ and autophagy‐related biomarkers in osteomyelitis

**DOI:** 10.1002/iid3.1339

**Published:** 2024-07-11

**Authors:** Xiangwen Shi, Mingjun Li, Haonan Ni, Yipeng Wu, Yang Li, Xianjun Chen, Yongqing Xu

**Affiliations:** ^1^ Kunming Medical University Kunming China; ^2^ Laboratory of Yunnan Traumatology and Orthopedics Clinical Medical Center Yunnan Orthopedics and Sports Rehabilitation Clinical Medical Research Center Kunming China; ^3^ Department of Orthopedic Surgery 920th Hospital of Joint Logistics Support Force of PLA Kunming Yunnan China; ^4^ Orthopedic Department First People's Hospital of Huzhou, First Affiliated Hospital of Huzhou University Huzhou China; ^5^ Department of Neurosurgery Nanping First Hospital Affiliated to Fujian Medical University Nanping Fujian China

**Keywords:** autophagy, clustering pattern, diagnosis, immune, immune infiltration, osteomyelitis

## Abstract

**Background:**

Osteomyelitis (OM) is recognized as a significant challenge in orthopedics due to its complex immune and inflammatory responses. The prognosis heavily depends on timely diagnosis, accurate classification, and assessment of severity. Thus, the identification of diagnostic and classification‐related genes from an immunological standpoint is crucial for the early detection and tailored treatment of OM.

**Methods:**

Transcriptomic data for OM was sourced from the Gene Expression Omnibus (GEO) database, leading to the identification of autophagy‐ and immune‐related differentially expressed genes (AIR‐DEGs) through differential expression analysis. Diagnostic and classification models were subsequently developed. The CIBERSORT algorithm was utilized to examine immune cell infiltration in OM, and the relationship between OM clusters and various immune cells was explored. Key AIR‐DEGs were further validated through the creation of OM animal models.

**Results:**

Analysis of the transcriptomic data revealed three AIR‐DEGs that played a significant role in immune responses and pathways. Nomogram and receiver operating characteristic curve analyses were performed, demonstrating excellent diagnostic capability for differentiating between OM patients and healthy individuals, with an area under the curve of 0.814. An unsupervised clustering analysis discerned two unique patterns of autophagy‐ and immune‐related genes, as well as gene patterns. Further exploration into immune infiltration exhibited notable variances across different subtypes, especially between OM cluster 1 and gene cluster A, highlighting their potential role in mitigating inflammatory responses by regulating immune activities. Moreover, the mRNA and protein expression levels of three AIR‐DEGs in the animal model were aligned with those in the training and validation data sets.

**Conclusions:**

From an immunological perspective, a diagnostic model was successfully developed, and two distinct clustering patterns were identified. These contributions offer a significant resource for the early detection and personalized immunotherapy of patients with OM.

## Graphical abstract

From an immunological perspective, we have developed a diagnostic model and two distinct clustering patterns, providing a valuable resource for the early diagnosis and personalized immunotherapy of osteomyelitis (OM) patients.

## 1 INTRODUCTION

Osteomyelitis (OM) is recognized as a complex challenge in orthopedics, characterized by prolonged, inflammatory, and destructive processes affecting the bone and surrounding tissues due to microbial infections.^1^, ^2^ *Staphylococcus aureus* is identified as the most prevalent cause, leading to forms of OM that can be acute or chronic, with about 30% of acute cases evolving into chronic conditions.^3^, ^4^ Current treatment strategies are focused on antimicrobial therapy and thorough debridement to combat these infections. However, complications such as antibiotic resistance and abscess formation increasingly impeding the effectiveness of these treatments as the disease advances.^5^ Therefore, early diagnosis and timely treatment to prevent the transition from acute to chronic OM are recognized as crucial components.

The clinical diagnosis of OM typically involves multifaceted approaches that includes assessing clinical signs, radiological imaging, and laboratory test results. Further clarification of the diagnosis can be achieved through bone lesion tissue biopsy and microbial culture.^6^ While X‐rays are used as the initial imaging choice, their sensitivity for detecting early‐stage OM is notably limited, which diminishes their diagnostic utility. Notably, radiographic positivity in pediatric OM is observed at approximately 2 weeks, whereas in adults, a longer timeline may be required. Magnetic resonance imaging (MRI), on the other hand, is regarded as the diagnostic gold standard in OM imaging, offering superior sensitivity for lesion detection in later stages; however, its cost is high and does not significantly improve treatment outcomes.^7^ Laboratory tests often begin with the identification of elevated serum C‐reactive protein (CRP) and erythrocyte sedimentation rate (ESR) levels,^8^, ^9^, ^10^ though these indicators are not specific to OM, necessitating the exclusion of other infectious causes. The definitive diagnosis is often reliant on on serum or bone tissue cultures but has certain limitations, such as the need for invasive debridement surgery for tissue biopsy and bacterial culture. Furthermore, clear evidence of bone tissue infection may be present in some patients, yet the microbial culture results may still be negative. In summary, currently, a straightforward and rapid diagnostic biomarker or method for OM is lacking due to these limitations.

The pathogenesis of OM involves complex immune and inflammatory responses, as highlighted by previous research.^11^ In the initial phase, *S. aureus* is successfully in invading host tissues, leveraging its immune evasion tactics and antibiotic resistance, notably through biofilm formation.^12^, ^13^ Furthermore, *S. aureus* can persist within phagocytes like macrophages, neutrophils, and endothelial cells by promoting internalization, thus sidestepping early innate immune responses.^14^, ^15^ Additionally, Risk factors such as diabetes, obesity, and immune deficiencies are known to significantly elevate the likelihood of OM.^16^ Patient stratification offers a pathway to hierarchical treatment, yet broad interindividual variability challenges the creation of a standardized treatment grading system. The advent of bioinformatics and sequencing technologies has ushered in an era of more precise and comprehensive molecular targeted therapies.^17^, ^18^ Consequently, the identification of biomarkers that offer diagnostic and stratification value from an immunological and molecular standpoint is crucial for advancing OM treatment strategies.

**Figure 1:**
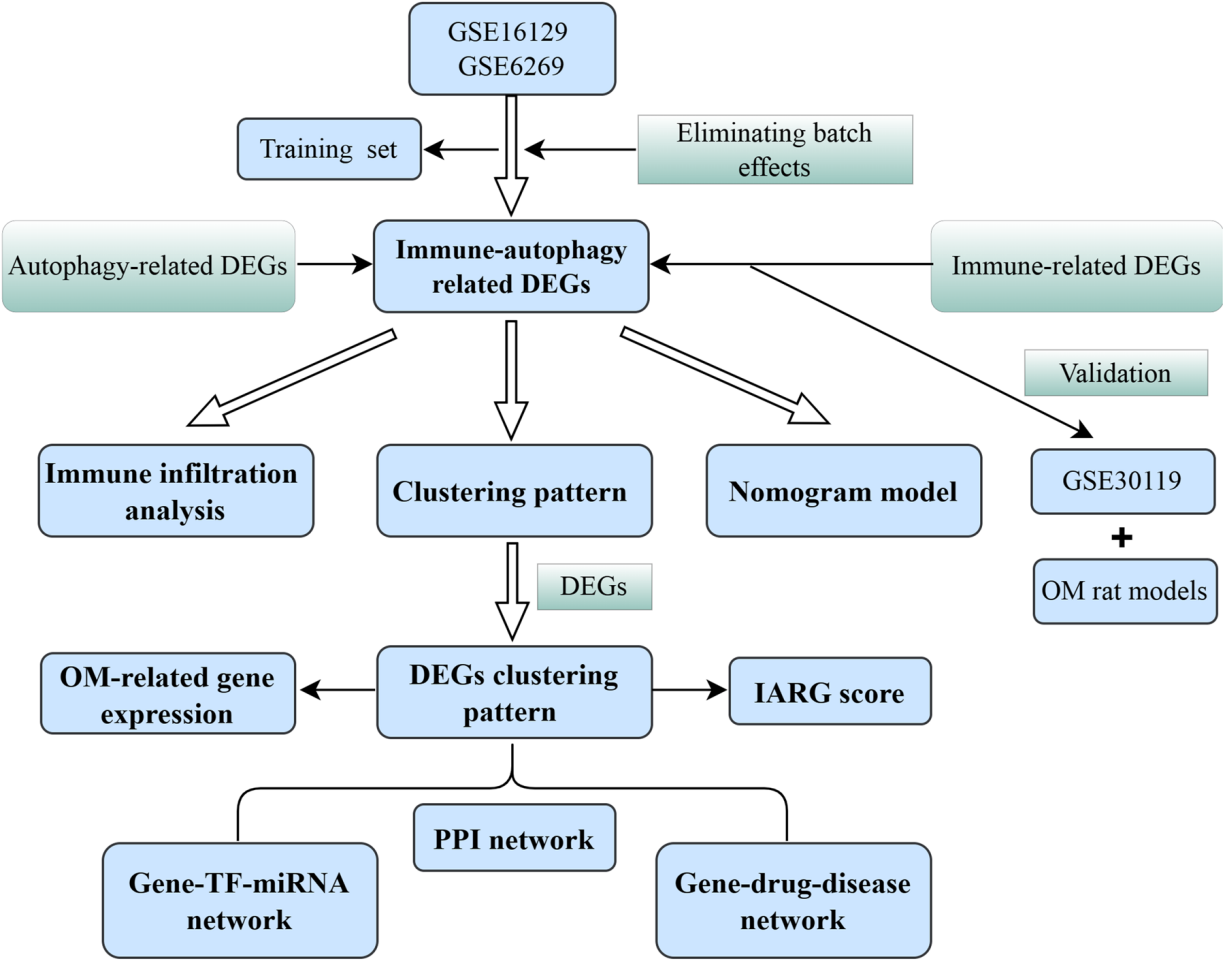
The workflow diagram of this study.

In this study, data from the Gene Expression Omnibus (GEO) and Immport databases were harnessed to identify differentially expressed genes (DEGs) linked to immune and autophagy. A nomogram model was then developed to assess the diagnostic potential of key genes in OM, with diagnostic accuracy of the model being evaluated by receiver operating characteristic (ROC) curve analysis. The reliability of model was further confirmed using an independent data set. Unsupervised clustering analysis was employed to classify OM patients into distinct molecular patterns, and the differences in immune cell infiltration between these patterns were explored. Moreover, rat models of OM were established to verify the expression levels of key genes. In conclusion, novel strategies for the diagnosis and individualized treatment of OM patients are provided by our diagnostic model and classification patterns (Figure 1).

## 2 MATERIALS AND METHODS

### 2.1 Data preprocessing

The gene expression data for three OM data sets (GSE16129, GSE6269, and GSE30119) and their corresponding platform files were downloaded from the GEO database (https://www.ncbi.nlm.nih.gov/geo/). The GSE6269 data set consists of 15 OM samples and 6 healthy samples and the GSE16129 data set includes 70 OM samples and 29 healthy samples. Two data sets were obtained from the GPL96 sequencing platform. The GSE30119 data set includes 39 OM samples and 44 healthy samples, obtained from the GPL6947 sequencing platform. Next, the combat function in the “sva” package^19^ was performed to merge the GSE16129 and GSE6269 data sets as a training set, which includes 85 OM samples and 35 healthy samples. The GSE30119 data set was used as a validation set to verify the consistency of the results in this study. Based on the foundation of previous research, we extracted 232 autophagy‐related genes (ARGs) and 2483 immune‐related genes (IRGs) as specific gene sets for further analysis from the Human Autophagy Database (http://www.autophagy.lu/index.html) and Immport database (https://www.immport.org/), respectively. The detailed information of these genes is provided in Supporting Information: Tables 1 and 2.

### 2.2 Identification of autophagy‐ and immune‐related DEGs (AIR‐DEGs)

Differential expression analysis was performed on 232 ARGs using the “limma” package^20^ to identify differentially expressed ARGs (DE‐ARGs) between OM samples and healthy control. Similarly, differential expression analysis was conducted on 2483 IRGs to identify differentially expressed IRGs (DE‐IRGs). Subsequently, differential heatmaps visualizing the DE‐ARGs and DE‐IRGs were generated using the “pheatmap” package.^21^ Intersection genes between DE‐ARGs and DE‐IRGs were further filtered. Additionally, correlation analysis and chromosomal localization analysis were performed on the intersection genes of the AIR‐DEGs. The filtering criteria were set with an adjusted *p*‐value < .05 and log‐fold change (FC) > |0.585|.

### 2.3 Protein–protein interaction (PPI) network analysis

To explore genes in OM that potentially interact physically with AIR‐DEGs, a PPI network was constructed using the String online platform (https://cn.string-db.org/). The obtained nodes and edges of the PPI network were visualized using Cytoscape software (version 3.9.1).^22^ To build the PPI network for AIR‐DEGs, a low confidence threshold of 0.15 was set.

### 2.4 Construction of transcription factors (TFs)–microRNAs (miRNAs) and gene–gene interaction networks

To further explore the potential molecular mechanisms of hub AIR‐DEGs, a regulatory TF–miRNA interactions network was constructed using the NetworkAnalyst online platform (https://www.networkanalyst.ca/). Specifically, a regulatory network associated with miRNA‐interacting characteristic genes was constructed based on the RegNetwork database. Additionally, a regulatory network linking characteristic genes and TFs was established using ChIP‐seq data from the ENCODE database, and a gene–gene interaction network was further constructed utilizing the GeneMANIA database.

### 2.5 Protein–drug and gene–disease interactions prediction

Information related to protein and drug targets was collected from the DrugBank database (version 5.0), and data associating hub AIR‐DEGs with human diseases was gathered from the DisGeNet database. The resulting interaction network was visualized using Cytoscape. All species considered in this study were human.

### 2.6 Diagnostic signature based on hub AIR‐DEGs

To predict the occurrence of OM, a nomogram model based on three hub AIR‐DEGs was constructed using the “rms” package. A clinical decision curves (DCA) was plotted to assess the clinical benefits of the nomogram model, and a calibration curve was used to evaluate the consistency between predicted values and observed values. Additionally, ROC curves were constructed to evaluate the diagnostic ability of hub AIR‐DEGs in OM patients. Furthermore, a logistic regression model for hub AIR‐DEGs was established using the “glmnet” package^23^ to predict whether a sample belongs to an OM patient or a healthy control. Finally, the GSE30119 data set was used as a validation set to verify the diagnostic capability of hub AIR‐DEGs in OM patients.

### 2.7 Identification of autophagy‐ and immune‐related genes (AIRG) patterns

Unsupervised clustering analysis based on three hub AIR‐DEGs enables the identification of distinct patterns among OM samples, which can aid in the stratified diagnosis and treatment of OM. First, the “ConsensusClusterPlus” package^24^ was employed to perform consistency clustering analysis on OM samples, with clustering parameters set as follows: max *K* = 9, reps = 50, pItem = 0.8, pFeature = 1. The optimal number of clusters was determined based on the results of the consensus heatmap and cumulative distribution function (CDF). Then, to assess significant differences between two AIRG patterns for different features, principal component analysis (PCA) was performed on the clustered OM samples, allowing further analysis of the significant differences in three hub AIR‐DEGs and the immune microenvironment between different patterns.

Additionally, DEGs were identified based on distinct AIRG clusters, and OM samples were further classified into different gene clusters. The parameter settings were consistent across the AIRG clusters. Furthermore, expression differences among the three hub AIR‐DEGs across the gene clusters were explored. Subsequently, the AIRG (cluster‐related gene) scores for each OM sample were evaluated using PCA and consistencies among AIRG cluster, gene cluster and AIRG score were compared. Finally, expression differences in genes related to the interleukin family, bone morphogenetic protein (BMP) family, and metalloproteinase in two different clusters (AIRG cluster and gene cluster) were analyzed.

### 2.8 Immune infiltration analysis in OM patients

The complex immune microenvironment mediated by OM may involve infiltration and regulation of various immune cells. The “CIBERSORT” algorithm^25^ was employed to assess the infiltration of immune cells in each OM and control group. Specifically, differences in the infiltration abundance of 22 immune cell types between OM and control samples were compared and visualized using histograms and boxplots. Subsequently, a correlation analysis was performed between the 3 hub AIR‐DEGs and the infiltration abundance of the 22 immune cell types. Furthermore, based on the median expression levels of each AIR‐DEG, samples were divided into high‐expression and low‐expression groups to explore differences in the proportions of immune cell infiltration between the two groups.

### 2.9 Clinical correlation analysis

To explore the association between the three hub AIR‐DEGs and clinical data of OM patients, clinical information (age and length of hospital stay) was extracted from the GSE6269 and GSE16129 data sets. A correlation analysis was then performed.

### 2.10 Expression of hub AIR‐DEGs in validation set

To validate the consistent differences or trends of hub AIR‐DEGs between OM and control samples, the gene expression data from the GSE30119 data set was utilized as a validation set. Specifically, the expression levels of the hub AIR‐DEGs were examined between 39 OM samples and 44 control samples.

### 2.11 Establishment of rat model of OM

Twelve Sprague–Dawley (SD) rats weighing 300–500 g were obtained from Kunming Chushang Co., Ltd., with six rats assigned to the experimental group and six rats to the control group, all of which were male. All SD rats were placed in the specific pathogen‐free environment of the Animal Experiment Center at 920 Hospital for 1 week of acclimatization feeding, with controlled temperature and lighting, without any restrictions on food and water. Rats were anesthetized by intravenous injection of pentobarbital sodium. After exposing the tibia, a Kirschner wire was inserted into the medullary cavity, and 20 μL of 1 × 10^6^ colony‐forming units of *S. aureus* (ATCC25923; Thermo Fisher Scientific Inc.) were injected into the tibial medullary cavity for the experimental group, while the sham surgery group received an injection of sterile saline. The specific steps for establishing the OM model were based on the protocol by Poeppl et al.^26^ and previous publication by our team.^27^ All experimental procedures were approved by the Nursing and Use of Experimental Animals Ethics Committee of the Joint Logistics Support Force 920 Hospital (2023‐007‐01). All procedures were carried out in accordance with the “Laboratory animal—Guideline for ethical review of animal welfare” and the animal care and use regulations of 920 Hospital.

### 2.12 RT‐qPCR validation

After establishing the OM model, total RNA was extracted from the tibial tissues of six rats in both the experimental and control groups. Reverse transcription was performed using the SweScrip RT I First Strand cDNA Synthesis Kit (ServiceBio). Following sample loading and brief centrifugation, the reaction was conducted for 40 cycles under the following conditions using the CFX96 Real‐Time Quantitative PCR System: initial denaturation at 95°C for 1 min, denaturation at 95°C for 20 s, annealing at 55°C for 20 s, and extension at 72°C for 30 s. The ethnographic primer sets used were as follows: BID: forward 5′‐AGCCGCTCCTTCTATCAT‐3′ and reverse 5′‐CAGGCAGTTCCTTTTGTC‐3′; CTSB: forward 5′‐CATTTGGGGCAGTGGAAG‐3′ and reverse 5′‐CAGGGAGGGATGGTGTAG‐3′; HSP90AB1: forward 5′‐TTGTATGTCCGTCGTGTGT‐3′ and reverse 5′‐CTCTGCTGGAGCATTTCTC‐3′; GAPDH: forward 5′‐GACCCCTTCATTGACCTCAAC‐3′ and reverse 5′‐GCCATCACGCCACAGCTTTCC‐3′.

### 2.13 Immunohistochemical staining

After extraction, tibial tissues from both the experimental and control groups were fixed in 4% paraformaldehyde and embedded in paraffin. Following dewaxing, the tibial tissues were incubated overnight at 4°C with primary antibodies against BID, CTSB, and HSP90AB1. The control tissues were incubated with sterile phosphate‐buffered saline (ServiceBio). Representative slices were selected and incubated with biotinylated secondary antibodies (ServiceBio) at 22°C. Following specimen sectioning, the staining levels of the three hub AIR‐DEGs in tibial tissues were observed under a microscope. Primary antibodies were as follows: BID (DF6016; Affinity Biosciences; 1:100), CTSB (14787‐1‐AP; Proteintech; 1:100), HSP90AB1 (BF0215; Affinity Biosciences; 1:200).

### 2.14 Statistical analysis

Student's *t*‐test was used for the comparison of differences between the two groups. Pearson's test was employed to reveal the relationship between hub AIR‐DEGs, immune infiltration, and patient clinical characteristics. *p* Value < .05 was considered statistically significant. Data analysis was performed using R software (version 4.1.3).

## 3 RESULTS

### 3.1 Identification of AIR‐DEGs in OM patients

**Figure 2:**
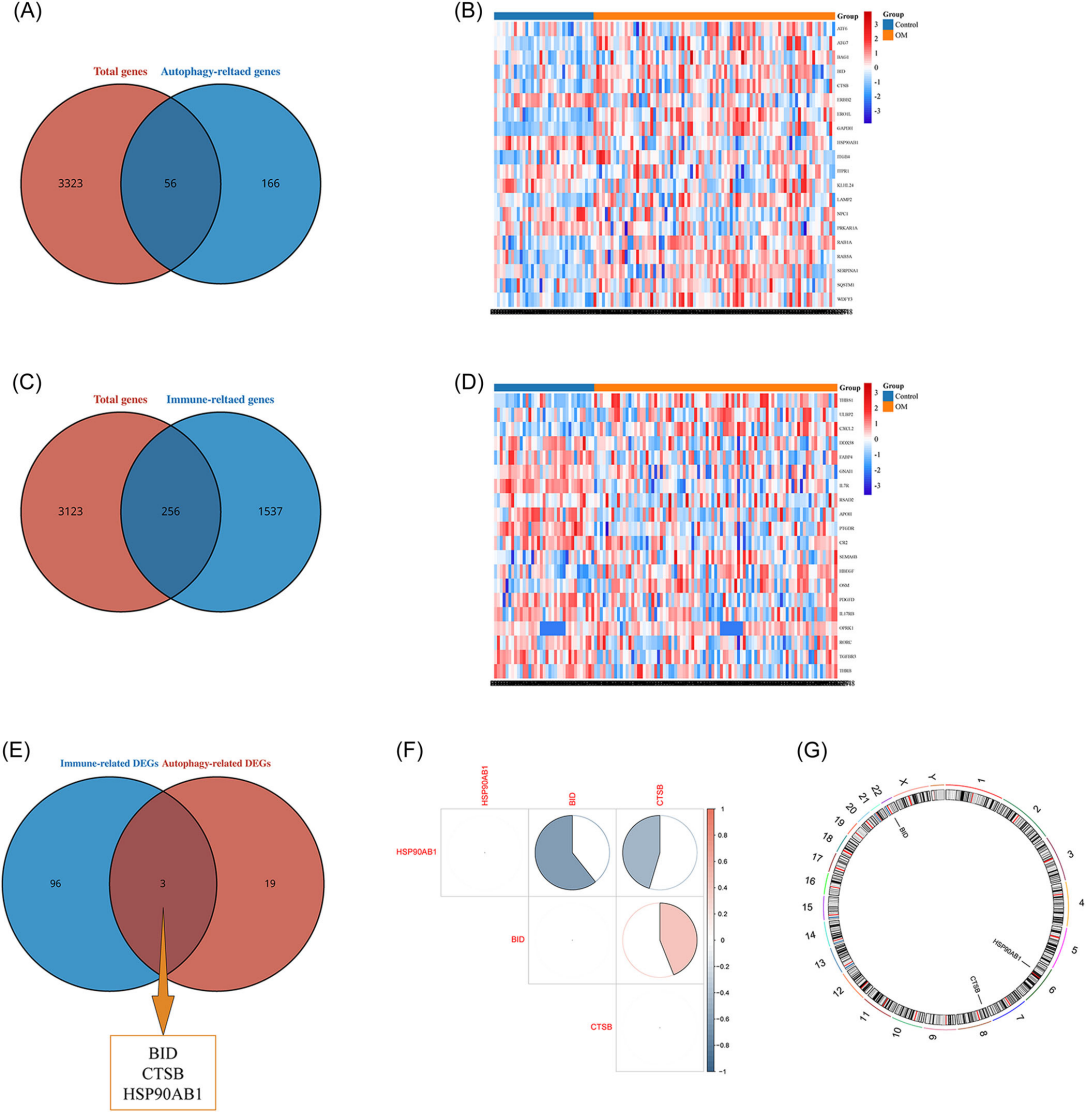
Screening of autophagy‐ and immune‐related differentially expressed genes (AIR‐DEGs). (A) Screening of autophagy‐related genes (ARGs). Red indicates the total genes, and blue indicates the ARGs. (B) Heatmap of AR‐DEGs. Red represents upregulated DEGs, and blue represents downregulated DEGs. (C) Screening of immue‐related genes (IRGs). Red indicates the total genes, and blue indicates the IRGs. (D) Heatmap of IR‐DEGs. Red represents upregulated DEGs, and blue represents downregulated DEGs. (E) Venn diagram of AIR‐DEGs. Red represents the AR‐DEGs, and blue represents the IR‐DEGs. (F) Correlation heatmap of three AIR‐DEGs. Red represents positive correlation, blue represents negative correlation. (G) Chromosome location of three characteristic genes.

After the data sets were merged and batch effects removed, 52 ARGs and 256 IRGs were identified from a total of 85 OM samples and 35 control samples (Figure 2A–D). Subsequent differential expression analysis revealed three AIR‐DEGs, and the results of correlation analysis showed a significant negative correlation between BID and CTSB with HSP90AB1. Conversely, a significant positive correlation was demonstrated between BID and CTSB (Figure 2E,F). The circular diagram illustrated the specific genomic positions of three AIR‐DEGs, with HSP90AB1, CTSB, and BID located on chromosomes 6, 8, and 22, respectively (Figure 2G).

### 3.2 Construction of PPI, TF–miRNA, and protein–disease networks

**Figure 3:**
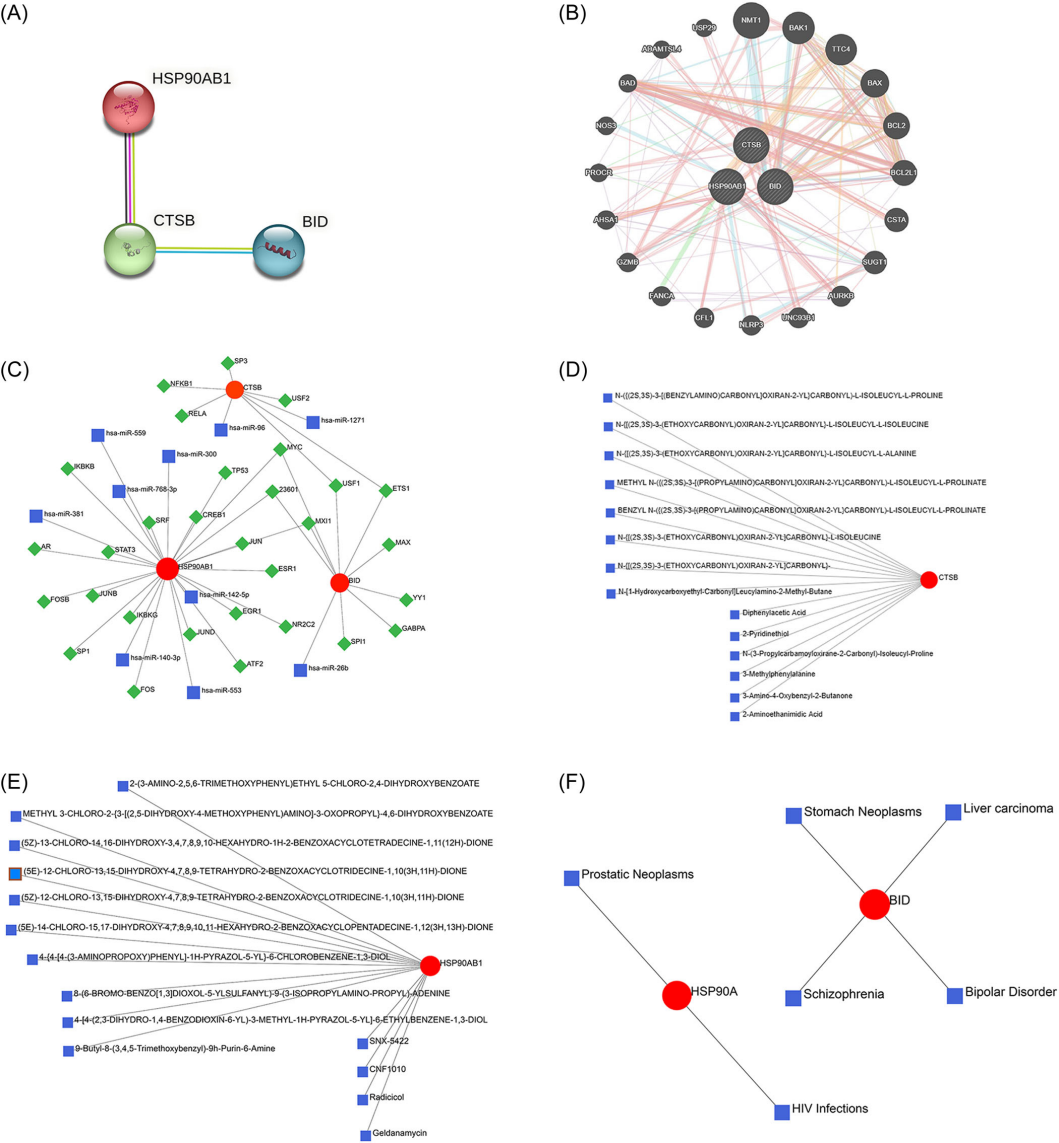
Construction of protein–protein interaction (PPI), gene–gene interaction and transcription factor (TF)–microRNA (miRNA) networks. (A) PPI network based on characteristic genes. (B) Gene–gene interaction network. (C) Gene‐TF‐miRNA network. (D) Gene–drug prediction based on CTSB. (E) Gene–drug prediction based on HSP90AB1. (F) Gene–disease network based on characteristic genes.

Utilizing the String online platform, a PPI network focused on BID, CTSB, and HSP90AB1 were constructed, showcasing interconnections of these genes (Figure 3A). Additionally, a gene–gene interaction network was developed through the GENMINIA online database, highlighting the top 20 interacting genes associated with the study identified (Figure 3B). To delve deeper into the underlying molecular mechanisms of the key AIR‐DEGs, gene–TF and gene–miRNA regulatory networks were established, uncovering 10 TFs and 30 miRNAs linked to these genes (Figure 3C). Furthermore, the therapeutic landscape was explored by predicting potential associations of CTSB and HSP90AB1 with 14 drugs, and possible connections of BID and HSP90AB1 to 4 and 2 human diseases, respectively, were mapped out, enhancing our understanding of their clinical relevance (Figure 3D–F).

### 3.3 Construction of OM prediction model based on hub AIR‐DEGs

**Figure 4:**
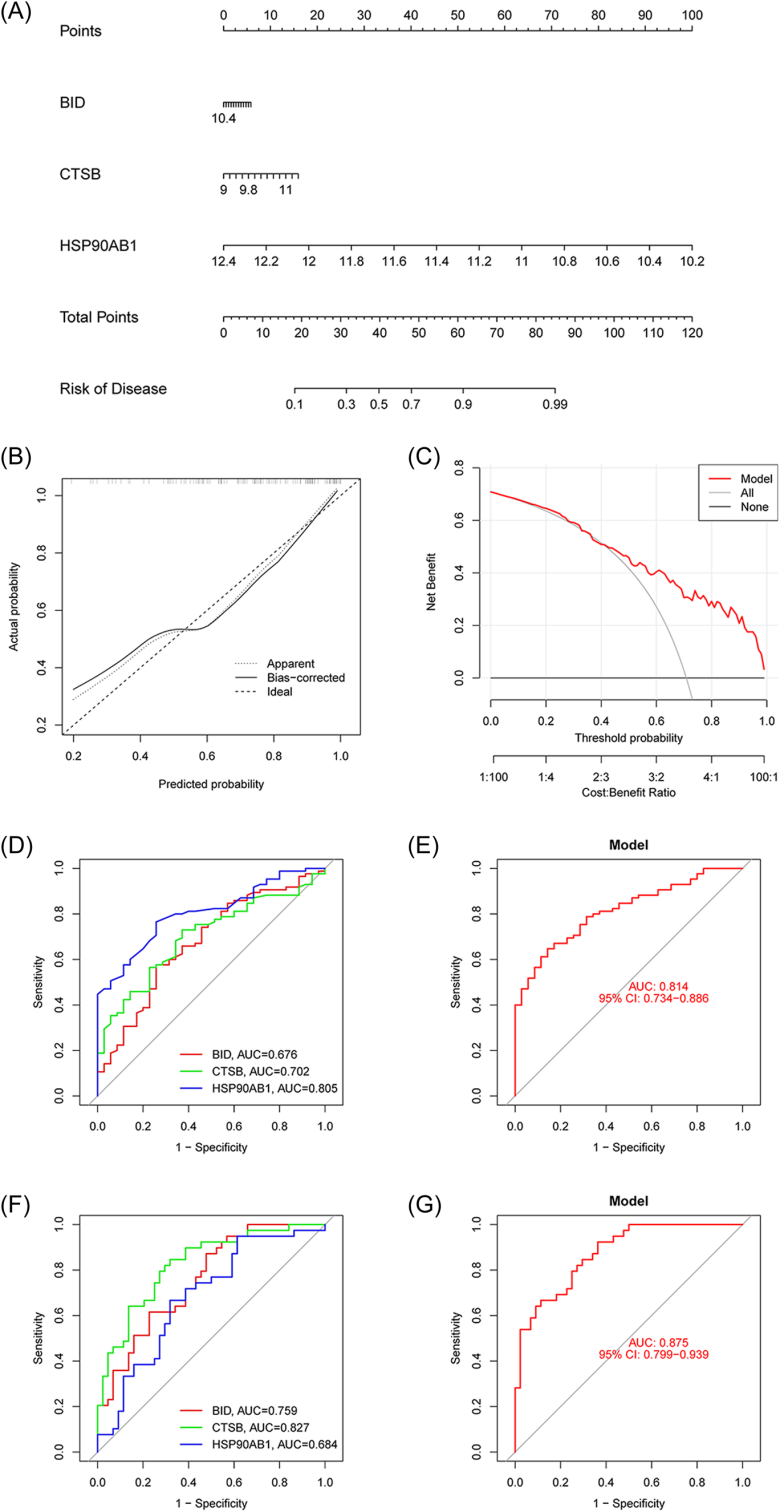
Diagnostic model and nomogram were identified for osteomyelitis (OM) patients based on three signature genes. (A) Establishment of nomogram model to predict the incidence of OM. (B) Calibration curve. (C) Clinical decision curve (DCA). (D) Receiver operating characteristic (ROC) curve of three signature genes in training set. (E). A logistic regression model based on three signature genes in training set. (F) ROC curve of three signature genes in validation set. (G) A logistic regression model based on three signature genes in validation set. ROC, receiver operating characteristic.

To forecast the occurrence of OM, a nomogram model utilizing the three identified AIR‐DEGs was developed. The predictive accuracy of model was assessed, demonstrating good outcomes (Figure 4A). A calibration curve further confirmed the effectiveness of model in predicting OM (Figure 4B), and DCA indicated that making clinical decisions based on this model could significantly benefit patients (Figure 4C). Additionally, the diagnostic value of the AIR‐DEGs in distinguishing OM patients was evaluated, with each gene achieving an area under the curve (AUC) value above 0.65, signifying robust diagnostic performance. Logistic regression analyses for these genes were performed using the “glmnet” package, and ROC curve analysis substantiated the capacity of model to accurately separate OM patients from healthy controls (AUC > 0.8) (Figure 4D,E). Validation through ROC curves, based on the GSE30119 data set, confirmed the potential of these three genes to differentiate between OM patients and healthy controls (Figure 4F,G), where all AIR‐DEGs exhibited AUC values exceeding 0.65, underscoring their effective diagnostic utility for OM.

### 3.4 Identification of AIRG patterns

**Figure 5:**
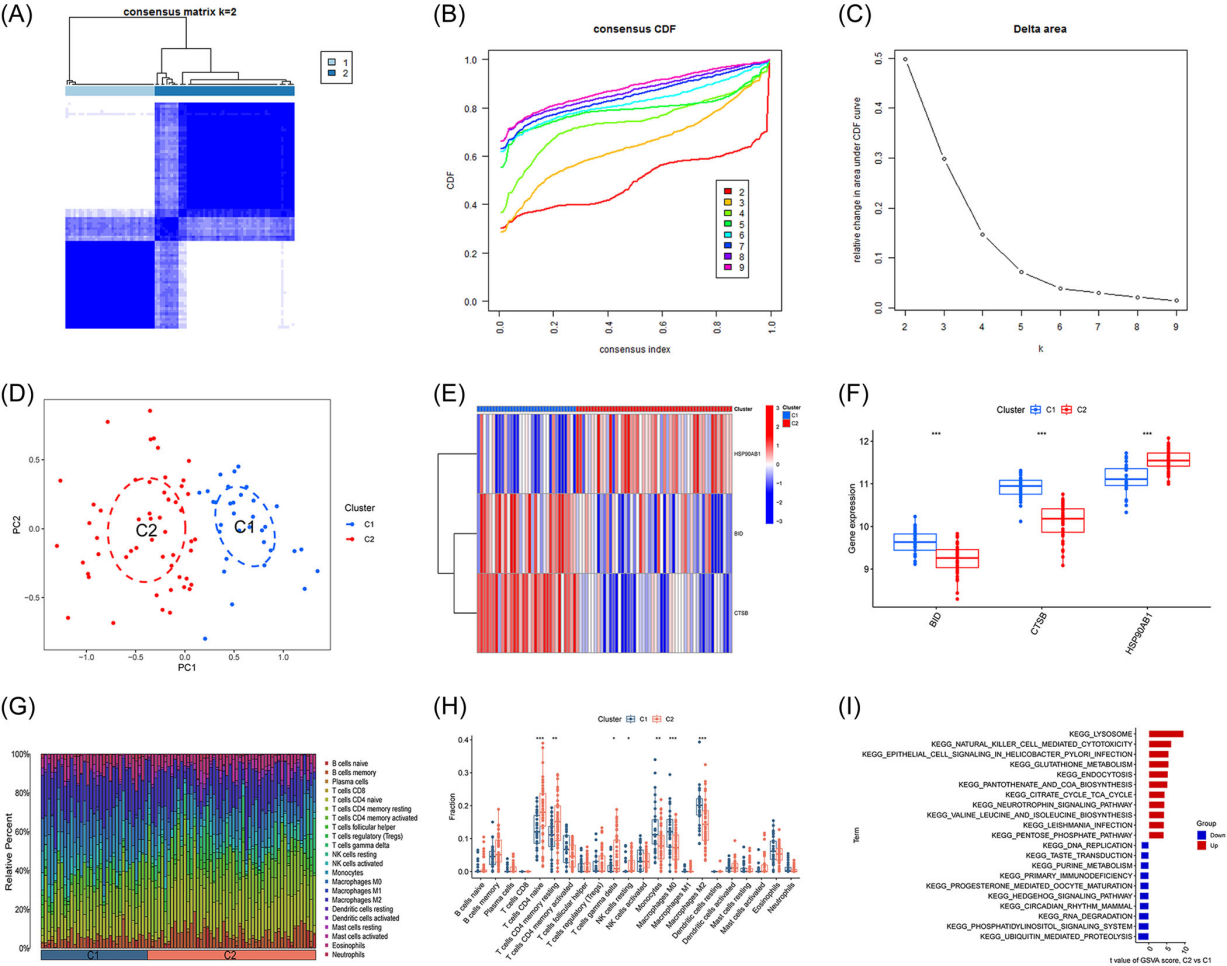
Identification of clusting pattern based on three signature genes. (A) Heatmap of two clusters (*k* = 2) based on signature genes. (B) Cumulative distribution function (CDF) of eight clusters. (C) The area under CDF curve of eight clusters. (D) Principal component analysis (PCA) analysis of the two clusters: blue indicates cluster 1 samples; red indicates cluster 2 samples. (E) Differential heatmap of three signature genes between two different cluster. Red represents high expression and blue represents low expression. (F) Differential boxplot of three signature genes between two different cluster. (G) Landscape of immune microenvironment between two different cluster. (H) Differential boxplot of infiltration abundance of 22 immune cell types between 2 different cluster. (I) GSVA‐KEGG pathway analysis between two different cluster (*p < .05; **p < .01; ***p < .001). GSVA, gene set variation analysis.

Employing unsupervised clustering analysis with the three pivotal AIR‐DEGs, distinct AIRG‐associated molecular patterns in OM patients were delineated (Figure 5A). Analysis of the CDF and AUC identified *K* = 2 as the optimal number of clustering subtypes, resulting with 33 samples in cluster 1 and 52 samples in cluster 2 (Figure 5B,C). PCA subsequently validated the significant variance in genetic features between these clusters (Figure 5D). Differential expression analysis revealed that BID and CTSB were more highly expressed in cluster 1, whereas HSP90AB1 showed higher expression in cluster 2 (Figure 5E,F). Further examination of immune cell infiltration variances between the two AIRG patterns unveiled significantly higher levels of naive CD4 T cells, resting CD4 memory T cells, gamma delta T cells, and resting NK cells in cluster 2, in contrast to increased infiltrations of monocytes, M0 macrophages, and M2 macrophages in cluster 1 (Figure 5G,H). Additionally, gene set variation analysis (GSVA)‐pathway analysis highlighted the upregulation of lysosome and NK cell‐mediated cytotoxicity pathways in cluster 2, while ubiquitin‐mediated proteolysis and primary immunodeficiency pathways were prominently upregulated in cluster 1 (Figure 5I). These insights suggested a potential role of cluster 1 in mediating anti‐inflammatory responses and cluster 2 in regulating NK cell activities within the context of OM.

### 3.5 Recognition of gene clusters and AIRG scores

**Figure 6:**
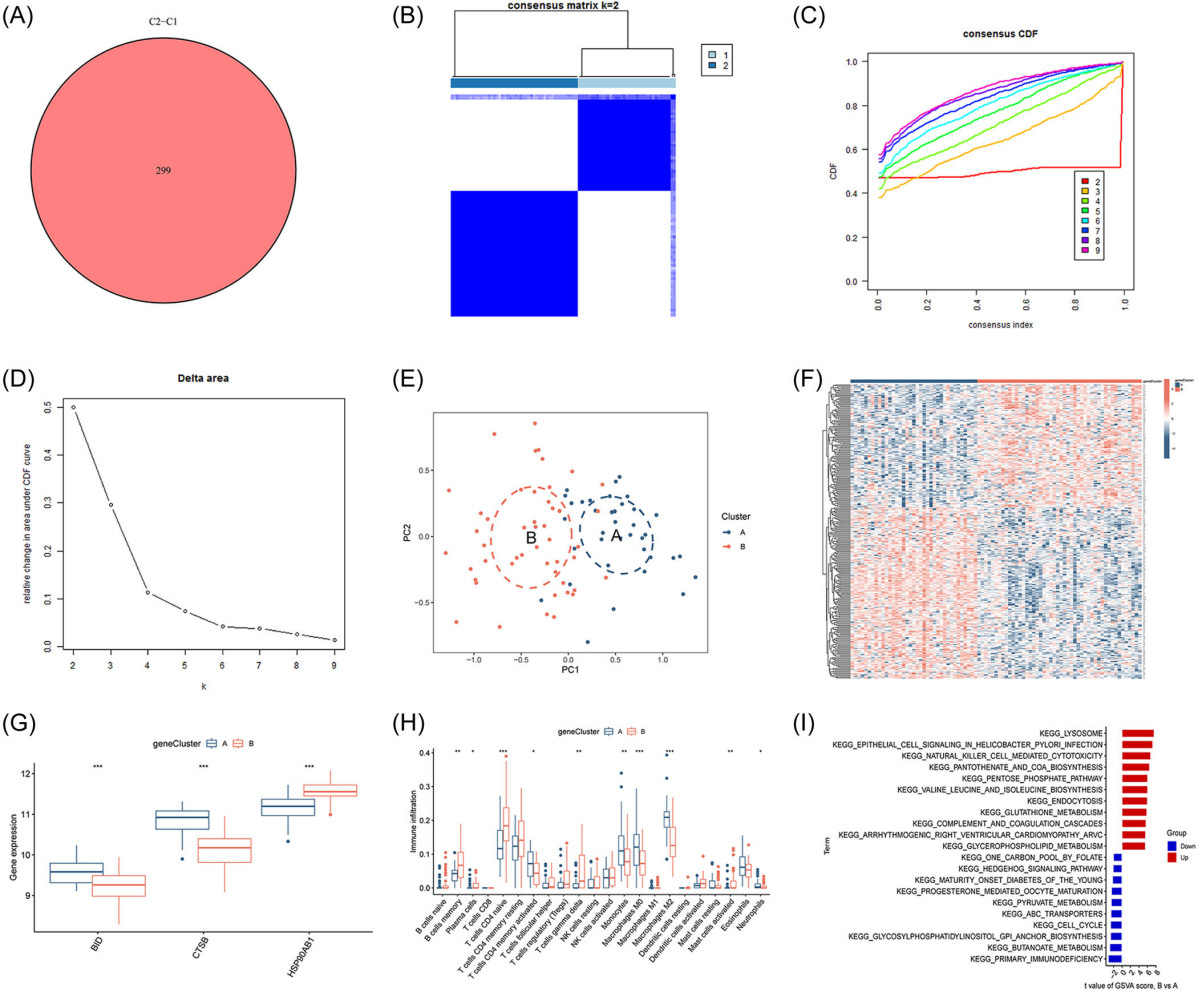
Identification of gene‐related clustering pattern. (A) Intersection‐DEGs between clusters 1 and 2. (B) Heatmap of two clusters (*k* = 2) based on signature genes. (C) Cumulative distribution function (CDF) of eight clusters. (D) The area under CDF curve of eight clusters. (E) Principal component analysis (PCA) analysis of the two clusters: blue indicates cluster A samples; red indicates cluster B samples. (F) Differential heatmap of 299 intersection‐DEGs between two different cluster. Red represents high expression and blue represents low expression. (G) Differential boxplot of three signature genes between two different cluster. (H) Differential boxplot of infiltration abundance of 22 immune cell types between two different cluster. (I) GSVA‐KEGG pathway analysis between two different cluster (*p < .05; **p < .01; ***p < .001). GSVA, gene set variation analysis.

To uncover the potential molecular mechanisms that differentiate AIRG subtypes, we identified 299 DEGs between OM cluster 1 and cluster 2 (Figure 6A). Subsequent analysis further divided the OM samples into distinct gene clusters using a clustering heatmap, the CDF curve, AUC, and PCA (Figure 6B–E). We determined that the optimal clustering subtypes were cluster A, containing 33 samples, and cluster B, with 52 samples, based on *K* = 2. Consistent with AIRG patterns, BID and CTSB were found to be more highly expressed in cluster A, whereas HSP90AB1 showed higher expression in cluster B (Figure 6F,G). Significant differences were noted in the immune microenvironment between the two gene clusters: Cluster B had elevated levels of memory B cells, plasma cells, naive CD4 T cells, gamma delta T cells, and activated mast cells, in contrast to cluster A, which exhibited higher proportions of activated CD4 memory T cells, monocytes, M0 and M2 macrophages, and neutrophils (Figure 6H). Further GSVA‐pathway analysis revealed a significant upregulation of the lysosome pathway in cluster B and the primary immunodeficiency pathway in cluster A (Figure 6I). These findings, aligning with previously identified AIRG patterns, suggest that cluster A may play a pivotal role in modulating the immune response in OM.

**Figure 7:**
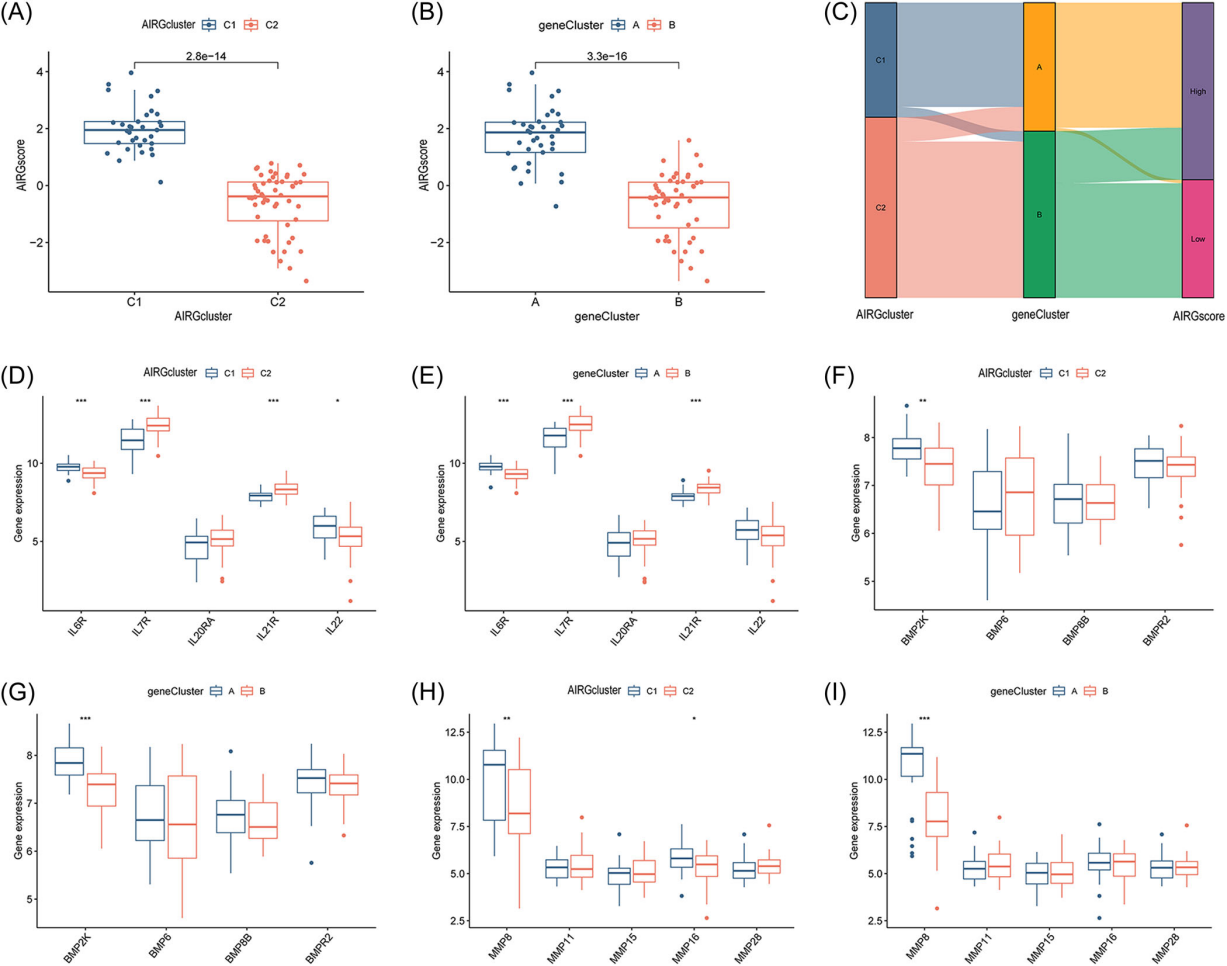
Autophagy‐ and immune‐related gene (AIRG)‐related cluster and gene‐related cluster. (A) AIRG score between two different AIRG‐related clusters. (B) AIRG score between two different gene‐related clusters. (C) Sankey diagram among AIRG‐related cluster, gene‐related cluster, and AIRG score. (D) Differential expression analysis of interleukin family‐related genes between two different AIRG‐related clusters. (E) Differential expression analysis of interleukin family‐related genes between two different gene‐related clusters. (F) Differential expression analysis of bone morphogenetic protein (BMP) family‐related genes between two different AIRG‐related clusters. (G) Differential expression analysis of BMP family‐related genes between two different gene‐related clusters. (H) Differential expression analysis of matrix metalloproteinase (MMP) family‐related genes between two different AIRG‐related clusters. (I) Differential expression analysis of MMP family‐related genes between two different gene‐related clusters (*p < .05; **p < .01; ***p < .001).

Based on the expression levels of DEGs between AIRG clusters and gene clusters, PCA was employed to generate scores (AIRG scores) for OM samples. The differences between the two clustering scores were compared (Supporting Information: Table 3). Within the internal comparisons of AIRG clusters and gene clusters, elevated AIRG scores were observed in AIRG cluster 1 and gene cluster A (Figure 7A,B). A Sankey diagram further illustrated the coherence among AIRG clusters, gene clusters, and AIRG scores, showcasing their interrelationship (Figure 7C). Differential expression analysis of pivotal genes revealed that IL7R and IL21R were more highly expressed in AIRG cluster 2 and gene cluster B, while IL6R exhibited higher expression in AIRG cluster 1 and gene cluster A (Figure 7D,E). Similarly, BMP2K and MMP8 were found to be highly expressed in both AIRG cluster 1 and gene cluster A (Figure 7F–I). These findings suggest that AIRG cluster 2 and gene cluster B may correspond to more intense inflammatory states, whereas AIRG cluster 1 and gene cluster A might play key roles in modulating the differentiation of osteoblasts and osteoclasts in OM.

### 3.6 Immune microenvironment analysis in OM patients

**Figure 8:**
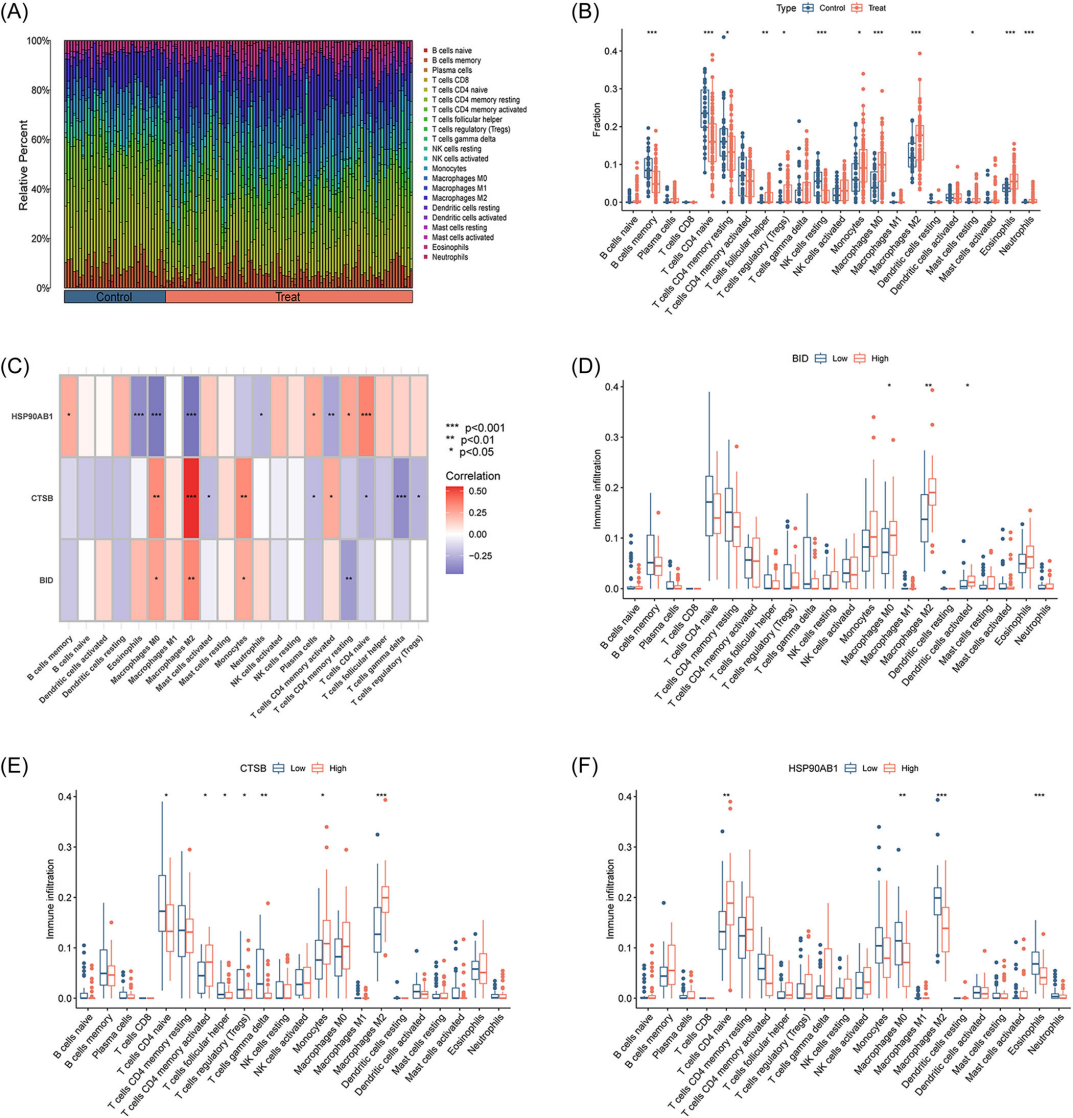
Immune microenvironment analysis of osteomyelitis (OM) patients. (A) Landscape of immune microenvironment between OM patients and healthy controls. (B) Differential boxplot of infiltration abundance of 22 immune cell types between OM patients and healthy controls. (C) Correlation heatmap of 3 signature genes and infiltration abundance of 22 immune cells. (D) The infiltration abundance of 22 immune cells between BID high‐ and low‐expression group. (E) The infiltration abundance of 22 immune cells between CTSB high‐ and low‐expression group. (F) The infiltration abundance of 22 immune cells between HSP90AB1 high‐ and low‐expression group (*p < .05; **p < .01; ***p < .001).

The pathophysiology of *S. aureus*‐induced OM is characterized by intricate immune mechanisms. To investigate the alterations in the immune microenvironment associated with OM, the “CIBERSORT” package was employed to calculate the infiltration proportions of 22 immune cell types in each OM and healthy sample. Significant differences in the proportions of these immune cell types were observed between the two groups, with eight immune cell types significantly upregulated in OM patients (Figure 8A,B). Furthermore, a correlation analysis between the 3 AIR‐DEGs and the proportions of the 22 immune cell types was conducted (Figure 8C). The results indicated a significant negative correlation between HSP90AB1 and M0 macrophages and M2 macrophages, while CTSB was found to have a significant positive correlation with M2 macrophages.

To explore the relationship between the expression levels of key AIR‐DEGs and the proportions of the 22 immune cell types, OM samples were divided into high‐ and low‐expression groups based on the median expression levels of the key AIR‐DEGs. It was shown that a significant correlation exists between high expression of BID and increased proportions of M0 macrophages, M2 macrophages, and activated dendritic cells (Figure 8D). In the CTSB high‐expression group, activated CD4 memory T cells, monocytes, and macrophages M2 were significantly upregulated, whereas naive CD4 T cells, follicular helper T cells, regulatory T cells (Tregs), and gamma delta T cells were significantly upregulated in the CTSB low‐expression group (Figure 8E). Higher infiltration proportions of M0 macrophages, M2 macrophages, and eosinophils were observed in the HSP90AB1 low‐expression group, while higher infiltration proportions of naive CD4 T cells were observed in the HSP90AB1 high‐expression group (Figure 8F). These results suggest that the upregulation of BID and CTSB may enhance the expression of M2 macrophages, contributing to an anti‐inflammatory effect. Conversely, the upregulation of HSP90AB1 may inhibit the expression of M2 macrophages in this process.

### 3.7 Clinical correlation analysis and expression levels of AIR‐DEGs in validation set

**Figure 9:**
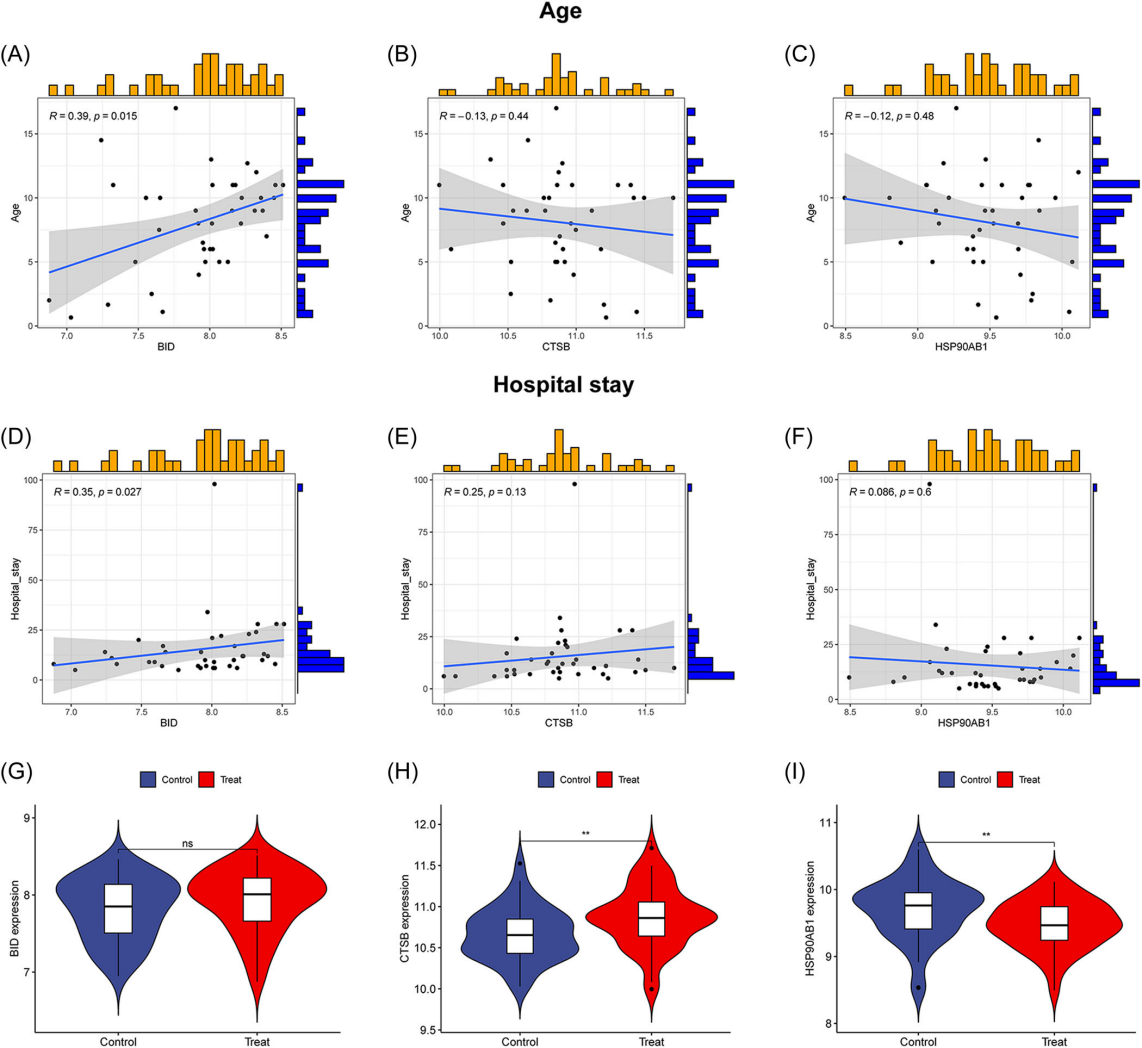
Clinical correlation analysis and expression levels of three autophagy‐ and immune‐related differentially expressed genes (AIR‐DEGs) in osteomyelitis (OM) patients. Correlation analysis between BID (A), CTSB (B), and HSP90AB1 (C) and age of OM patients. Correlation analysis between BID (D), CTSB (E), and HSP90AB1 (F) and hospital stay of OM patients: R > 0 indicates the two variables are positively correlated; R < 0 indicates the two variables are negatively correlated; *p* < .05 represents that the two variables are significantly correlated. Expression levels of BID (G), CTSB (H), and HSP90AB1 (I) in the validation set (ns: *p* ≥ .05, no significant difference; **p < .01).

To assess the clinical significance of the three AIR‐DEGs in OM patients, age and hospitalization duration data from the OM patient cohort were analyzed. A significant positive correlation between BID and the age of OM patients was revealed through correlation analysis (Figure 9A), while no significant correlations were found for CTSB and HSP90AB1 (Figure 9B,C). Consistent with the age correlation, a positive correlation was also observed between BID and the duration of hospitalization in OM patients (Figure 9D), with no significant correlations noted for CTSB and HSP90AB1 (Figure 9E,F). The elevated expression of BID was associated with older age and longer hospital stays in OM patients, suggesting a more severe OM condition. Additionally, the expression levels of the three AIR‐DEGs were validated using an independent data set, GSE30119. Although no significant difference in expression levels of BID was shown in the validation set, the expression trend remained consistent with the training set (Figure 9G). CTSB was found to be significantly upregulated in OM samples, while HSP90AB1 was shown to be downregulated (Figure 9H,I).

### 3.8 RT‐qPCR and immunohistochemistry validation

**Figure 10:**
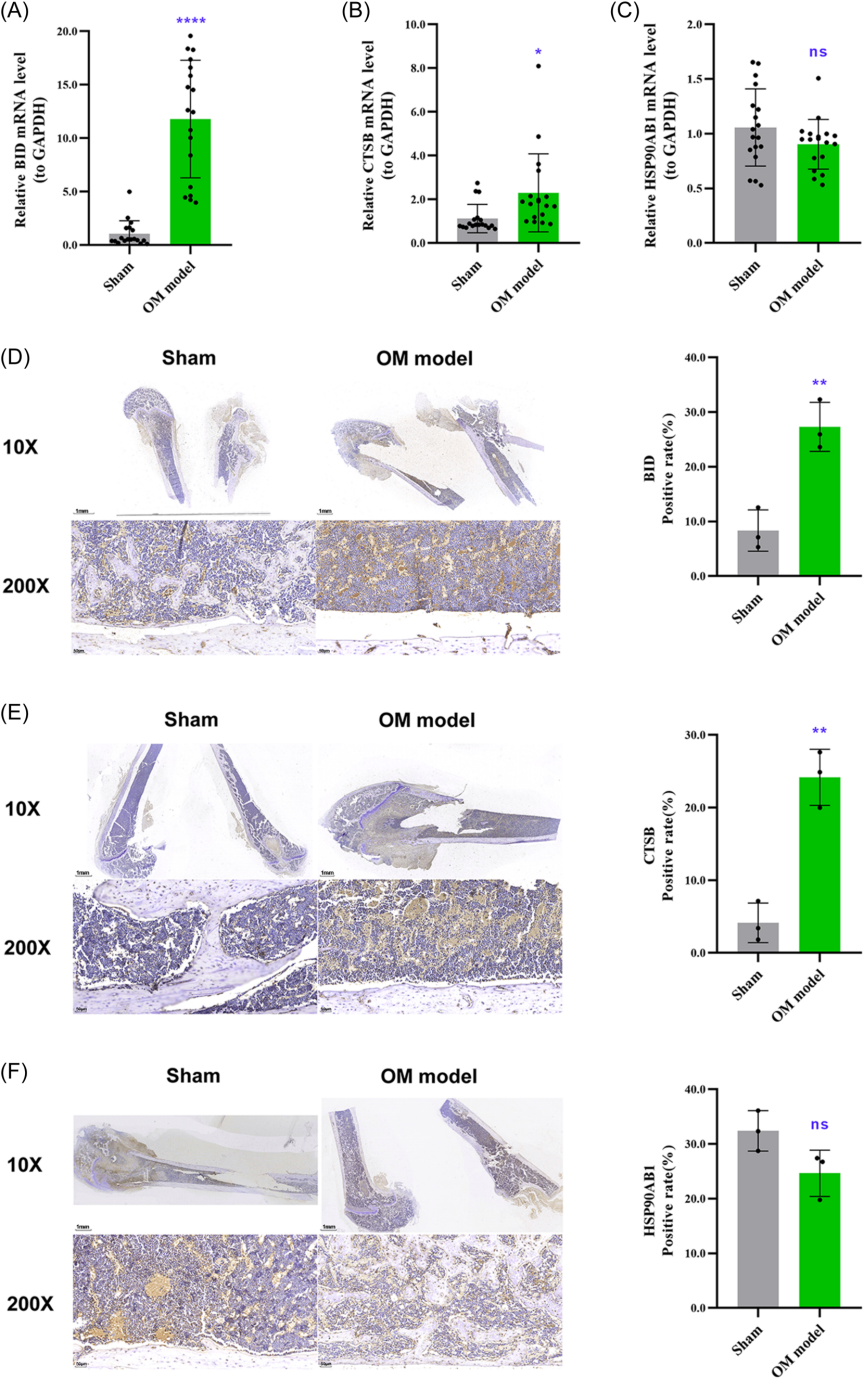
RT‐qPCR and immunohistochemistry validation. Following the establishment of rat models of osteomyelitis (OM), total RNA was extracted from the focal bone tissues of the OM group and the sham group. The mRNA expression levels of BID (A), CTSB (B), and HSP90AB1 (C) were measured. Immunohistochemical staining was performed on the fixed tibial tissues to evaluate the protein expression levels of BID (D), CTSB (E), and HSP90AB1 (F), with the microscopic positivity rate serving as the measurement index. ns: *p* ≥ .05, no significant difference; *p < .05; **p < .01; ****p < .001.

Further RT‐qPCR analysis revealed that the mRNA levels of BID and CTSB were significantly upregulated in the OM group compared to the sham group (Figure 10A,B). No significant difference in the mRNA level of HSP90AB1 between two groups, but its expression trend was consistent with the training set and validation set (Figure 10C). Finally, immunohistochemistry was employed to examine the protein expression levels of three AIR‐DEGs. Significantly higher staining positivity rates of BID and CTSB were observed in OM group compared to sham group (Figure 10D,E), while HSP90AB1 exhibited a decreasing trend in positivity rate in the OM group (Figure 10F), aligning with the bioinformatics results.

## 4 DISCUSSION

OM is an inflammatory bone disease caused by microbial infections, where bacteria or fungi aggressively multiply after breaching the bloodstream or bone tissue through surgery or trauma.^28^ In the United States, the annual incidence of OM is reported at 2.8 cases per 100,000 individuals, and its frequent recurrence and persistent nonhealing impose a significant economic burden.^29^, ^30^ The diagnosis of OM traditionally hinges on clinical observations, imaging data, and lab tests, with invasive procedures like puncture or biopsy being required for confirmation.^31^, ^32^ Early detection is recognized as paramount for better outcomes in OM patients, as delays can lead to severe complications, including deformities and sepsis.^33^ However, conventional X‐ray and MRI examinations are limitations by a low positivity rate and time lag, while CRP and ESR are recognized as nonspecific indicators of OM.^34^, ^35^ Furthermore, the selection of imaging techniques for OM diagnosis is also influenced by various factors such as infection location, presence of metal implants, infection duration, antibiotic usage, and complications.^36^ Therefore, an urgent need for a novel diagnostic approach is recognized to address these challenges. Moreover, the invasion by OM‐causing microbes, notably *S. aureus*, involves intricate immune responses, yet the specific mechanisms of regulation remain to be fully understood. In recent years, the rapid advancement of bioinformatics and various machine learning models has made molecular diagnosis and targeted therapy possible.^37^, ^38^ We aim to explore a specific biomarker that integrates immune and molecular insights for the rapid diagnosis of OM patients.

In this study, a biological approach was employed to screen and identify core genes related to immunity and autophagy, ultimately leading to the creation of a diagnostic and clustering model of OM. Detailed data on OM patients, IRGs, and ARGs were gathered from publicly accessible databases. Differential expression analysis was utilized to screen for intersecting genes pertinent to both immunity and autophagy, which were designated as core AIR‐DEGs. Further investigations delved into the interaction networks and potential biological roles of these core genes, encompassing gene–gene, protein–protein, and TF–miRNA interaction networks. It has been demonstrated in previous studies that bone tissue destruction in the context of OM is attributed to reduced bone matrix synthesis and increased osteoblast apoptosis, leading to subsequent bone loss.^39^ From a pathogenic mechanism perspective, osteoblast cell death has been induced by *S. aureus* through apoptosis, necrotic apoptosis, and cellular autophagy, and its major virulence protein, SpA, has been shown to induce cell apoptosis and participate in the pathogenesis of OM.^40^, ^41^, ^42^ Consistent with the findings of this study, three AIR‐DEGs were found to be associated with antigen processing and presentation, cellular apoptosis, and programmed cell death signaling pathways, suggesting their potential roles in the pathogenic mechanism of OM through modulating immune responses and programmed cell death.

To further explore the diagnostic value of core genes in OM, a nomogram and ROC diagnostic model utilizing three AIR‐DEGs were developed. The model demonstrated good diagnostic performance for OM with an overall AUC of 0.814, and individual gene AUC values each exceeding 0.65. Its diagnostic accuracy was further validated using an independent data set, where it demonstrated excellent diagnostic capability (AUC = 0.875). In previous OM diagnostic models, good discriminatory ability for OM patients was demonstrated by ROC curves based on ferroptosis‐related genes (AUC = 0.993).^18^ In standard clinical practice, ESR and CRP are served as prevalent serological markers for OM, yet their specificity is limited. It was indicated by a study that the AUC values for OM diagnosis based on ESR and CRP were approximately 0.829 and 0.767, respectively. In comparison, the diagnostic performance of serum extracellular vesicles, although slightly lower at an AUC of 0.722, boasts a remarkable specificity of 93.3%.^43^ The efficacy of the three AIR‐DEGs in discriminating OM patients from healthy controls was highlighted by these findings, underscoring their promise as novel diagnostic biomarkers for OM.

The variability in patient demographics, medical histories, and clinical presentations contributes to the considerable challenges in establishing universally accepted classification guidelines for OM. Current classification schemes, such as distinguishing between acute and chronic OM, often rely on subjective criteria.^44^, ^45^, ^46^ Although chronic OM is traditionally considered to persist for 6 months or more,^47^ cases presenting within 4 weeks with evidence of lymphocyte infiltration and reactive new bone formation have been documented.^48^ Therefore, the determination of treatment based solely on the duration lacks scientific evidence. Furthermore, while imaging assessments play a pivotal role in OM classification, their utility is notably diminished in mild infections or when metallic implants interfere with image quality.^49^, ^50^ In response to these classification dilemmas, an innovative OM classification model utilizing three AIR‐DEGs was introduced in our study, categorizing OM patients into two distinct clusters: OM cluster 1 and cluster 2. These clusters were noted to exhibit significant differences in immune cell infiltration levels, with OM cluster 1 potentially mediating anti‐inflammatory effects through immune response modulation, and cluster 2 possibly influencing NK cell regulation in OM. These findings proposed that the identified AIR‐DEGs not only offered a novel approach for OM classification but also hinted at their involvement in the OM pathogenesis via modulation of immune and inflammatory responses.

Given the complex etiology of OM, which can be caused by various microbial infections or induced by noninfectious factors such as chronic nonbacterial osteomyelitis, animal model studies are recognized as crucial for the pathophysiological research of OM.^51^ In this study, femoral OM model in SD rats was established, and the potential of AIR‐DEGs as biomarkers was comprehensively assessed. The mRNA and protein expression levels of three key AIR‐DEGs within the bone lesions were specifically measured. The results indicated that the expression levels of BID and CTSB were significantly higher in the OM model group compared to the control group, while HSP90AB1 showed a trend towards lower expression. Notably, BID, which plays a role in the apoptotic signaling pathway, was previously identified by Chen et al.^52^ as a gene that could induce osteoblast apoptosis in the OM setting, possibly through a mechanism involving vitamin D deficiency. Additionally, the heat shock protein 90β (HSP90Β), encoded by HSP90AB1, has been found to promote the expression of osteoclasts through the NF‐κB signaling pathway, and genetic deletion of HSP90AB1 has been shown to alleviate bone loss in rats with osteoporosis.^53^ These findings suggest that BID and HSP90AB1 may serve as potential therapeutic targets for OM, warranting further functional studies.

Nevertheless, this study has certain limitations. First, despite the development of a well‐performing diagnostic model, it still requires validation through large‐sample and multicenter clinical trials. Second, to comprehensively validate the expression and functional roles of the identified genes, the utilization of additional OM models involving different animal species, such as mice and rabbits, is imperative. Third, only the mRNA and protein expression levels of key biomarkers in animal models were explored in this study. More in‐depth phenotypic and mechanistic experiments are needed to further investigate the specific mechanisms by which key AIR‐DEGs regulate OM immune and inflammatory responses.

## 5 CONCLUSION

In conclusion, our comprehensive analysis of three key AIR‐DEGs in OM has enabled the establishment of a robust diagnostic and classification model. This model provides valuable insights from an immunological perspective for the stratified diagnosis and graded treatment of patients with OM.

## AUTHOR CONTRIBUTIONS

Xiangwen Shi and Yongqing Xu conceived the study and wrote the manuscript. Mingjun Li and Haonan Ni carried out the data collection and data analysis. Xianjun Chen, Yang Li, and Yipeng Wu contributed to the data curation, methodology, and validation. All authors reviewed the results and approved the final version of the manuscript.

## CONFLICT OF INTEREST STATEMENT

The authors declare no conflict of interest.

## ETHICS STATEMENT

920th Hospital of Joint Logistics Support Force Committee on Ethics approved this study and consented to participate (2023‐007‐01).

## Supporting information

Supporting information.

Supporting information.

Supporting information.

## Data Availability

The microarray data used to support the findings of this study can be downloaded from the GSE6269, GSE16129, and GSE30119 data sets (https://www.ncbi.nlm.nih.gov/geo).
