# Supplementary material for: Integrative gene expression analysis and animal model reveal immune‐ and autophagy‐related biomarkers in osteomyelitis
Source: Immun Inflamm Dis. 2024 Jul 11;12(7):e1339. doi: 10.1002/iid3.1339 (PMC11238574; doi:10.1002/iid3.1339)
Supplement: Supplementary file 3 — Supporting information. [file IID3-12-e1339-s003.docx]

| Supplementary Table 3. AIRG scores through principal component analysis. | |
| --- | --- |
| id | AIRG score |
| GSM403287_treat | 2.144009 |
| GSM403293_treat | 1.722991 |
| GSM403295_treat | 1.864802 |
| GSM403297_treat | 0.710247 |
| GSM403299_treat | 2.225505 |
| GSM403301_treat | -0.44241 |
| GSM403307_treat | 1.668881 |
| GSM403309_treat | 0.120666 |
| GSM403311_treat | -0.3429 |
| GSM403315_treat | 0.424724 |
| GSM403319_treat | 0.395499 |
| GSM403321_treat | -0.16382 |
| GSM403394_treat | 1.587827 |
| GSM403398_treat | 0.134587 |
| GSM403404_treat | -0.40612 |
| GSM403406_treat | -2.3194 |
| GSM403410_treat | 0.071819 |
| GSM403414_treat | 3.360527 |
| GSM403418_treat | -1.95972 |
| GSM403420_treat | 0.119443 |
| GSM403422_treat | 1.94944 |
| GSM403425_treat | -0.54165 |
| GSM403428_treat | 2.049463 |
| GSM403430_treat | -3.35466 |
| GSM403434_treat | 0.307511 |
| GSM403436_treat | -2.91065 |
| GSM403438_treat | -2.65538 |
| GSM403442_treat | 1.41001 |
| GSM403549_treat | -1.79347 |
| GSM173178_treat | -1.10315 |
| GSM173182_treat | 2.099321 |
| GSM173187_treat | 3.322833 |
| GSM173193_treat | -2.0051 |
| GSM173196_treat | 2.621683 |
| GSM173230_treat | 1.130571 |
| GSM173256_treat | 0.116078 |
| GSM173260_treat | -0.03534 |
| GSM173271_treat | -0.30799 |
| GSM173275_treat | -0.52307 |
| GSM173204_treat | -0.35568 |
| GSM173249_treat | 0.634555 |
| GSM173251_treat | 2.073699 |
| GSM173253_treat | 0.294315 |
| GSM173254_treat | 1.921517 |
| GSM403550_treat | -0.20195 |
| GSM403560_treat | -1.94301 |
| GSM403561_treat | 0.873548 |
| GSM403571_treat | 1.58296 |
| GSM403572_treat | -1.93971 |
| GSM403573_treat | -0.73007 |
| GSM403574_treat | 3.555981 |
| GSM403575_treat | 2.045854 |
| GSM403286_treat | 1.475159 |
| GSM403292_treat | 0.593028 |
| GSM403294_treat | 2.245524 |
| GSM403296_treat | 0.37057 |
| GSM403298_treat | 2.213551 |
| GSM403300_treat | -0.01832 |
| GSM403306_treat | 2.510271 |
| GSM403308_treat | 0.497196 |
| GSM403310_treat | 0.378228 |
| GSM403314_treat | 0.0143 |
| GSM403318_treat | 0.780931 |
| GSM403320_treat | -0.49307 |
| GSM403393_treat | 0.094616 |
| GSM403397_treat | -0.73217 |
| GSM403403_treat | 1.077946 |
| GSM403405_treat | -1.19472 |
| GSM403409_treat | 1.280404 |
| GSM403413_treat | 3.961424 |
| GSM403417_treat | -1.84572 |
| GSM403419_treat | 1.163214 |
| GSM403421_treat | 2.478877 |
| GSM403423_treat | -0.42739 |
| GSM403424_treat | -0.64845 |
| GSM403426_treat | 1.505042 |
| GSM403427_treat | 1.27381 |
| GSM403429_treat | -2.32889 |
| GSM403431_treat | 0.166447 |
| GSM403432_treat | -0.09108 |
| GSM403433_treat | -0.59707 |
| GSM403435_treat | -1.38459 |
| GSM403437_treat | -2.33576 |
| GSM403441_treat | 3.136669 |
| GSM403548_treat | -0.67544 |
